# Editorial: CONSTANS – signal integration and development throughout the plant kingdom

**DOI:** 10.3389/fpls.2024.1375876

**Published:** 2024-02-20

**Authors:** Fabio T. S. Nogueira, Daniela Goretti, Federico Valverde

**Affiliations:** ^1^ Laboratory of Molecular Genetics of Plant Development, Escola Superior de Agricultura “Luiz de Queiroz” (ESALQ), University of São Paulo (USP), Piracicaba, São Paulo, Brazil; ^2^ Faculty of Science and Technology, Department of Plant Physiology, Umeå Plant Science Centre (UPSC), Umeå University, Umeå, Sweden; ^3^ Plant Development Group - Institute for Plant Biochemistry and Photosynthesis, Consejo Superior de Investigaciones Científicas, Universidad de Sevilla, Seville, Spain

**Keywords:** CONSTANS, photoperiod, evolution, diversity, BBX transcription factor, metabolomics, transcriptomics

In the past decades, several findings have demonstrated that plants and animals employ a complex network of inducing and inhibiting pathways integrating environmental and internal signals to monitor the proper timing for developmental and physiological processes. Among the environmental signals with a major effect on plants and animals, daylength is considered the most robust (Bradshaw and Holzapfel, 2007; Gendron and Staiger, 2023). The main mechanism that senses daylength includes the photoperiod pathway, which is an ancient regulatory system found from microalgae to vascular plants, and constitutes one of the earliest examples of developmental regulation (Serrano-Bueno et al., 2021).

The photoperiod signaling mechanism is present in all known plant species (Serrano-Bueno et al., 2017), but it is best known as the inductor of flowering in the model plant *Arabidopsis thaliana*. Light, along with daylength and the circadian clock, channels molecular information through the photoperiod pathway to modulate its central regulator, the BBX transcription factor CONSTANS (CO). CO belongs to the CCT [CO, CO-like, and TIMING OF CAB EXPRESSION1 (TOC1)] domain-containing protein family, and acts as a “hub” of light and clock inputs to trigger the expression of the mobile florigen *FLOWERING LOCUS T* (*FT*) in leaves, what is known as the ‘coincidence model’ for flowering (Valverde et al., 2004).

Although the roles of CCT family members have been intensively studied in flowering plants such as *A. thaliana*, rice, and maize, little is known about the roles of *CO-like* genes in other species. Hence, an extensive effort is needed to unravel possible new functions of CCT family members to favor a more comprehensive scenery of how plants incorporate daylength information into their physiology. This Research Topic represented the collection of four original research articles expanding our understanding of how nonconventional model plants integrate environmental signals and physiology.

The research articles discussed in this Research Topic mostly focus on the identification of new *CO-like* genes, which could assist in unraveling overlooked photoperiodic-dependent mechanisms during evolution. Gene duplication is one of the major mechanisms to supply raw genetic material for evolution, and it may influence how species cope with environmental modifications throughout evolution. For instance, duplicated regulators and their targets can acquire mutations that lead to gradual increases in specificity, allowing neofunctionalization or subfunctionalization. The CCT family seemed to be subjected to these evolutionary modifications as shown by Li et al. in their study. In the foxtail millet, the CCT family could expand through duplication, while the duplicated genes possibly diverge over time to perform different functions. One of these CCT genes is the *SiPRR37*, homologous to the circadian rhythm-associated *PRR7* gene in *Arabidopsis*. *SiPRR37* was shown to be functionally associated with the control of flowering time (heading date) in foxtail millet. The study done by Liu et al. expands our knowledge regarding the control of flowering time and stress responses in mango by *CO-like* genes. The authors showed that, in addition to controlling flowering time, two mango *COL* genes can improve the tolerance of *Arabidopsis* plants growing under salt and drought conditions by increasing the reactive oxygen species (ROS) clearance ability.

The comprehensive study by Liang et al. sheds some light on the molecular mechanisms underlying the differences in flowering intensity among mango varieties. By investigating the transcriptome and metabolome profiles of flower-related tissues in three mango varieties, they found that gibberellin and auxin, sugar biosynthesis, and ambient temperature pathways interact with major flowering-associated genes (including *CO*) to modulate flowering intensity. Finally, Schmidt et al. identified *COL* genes in the day-neutral *Nicotiana tabacum* that do not affect flowering transition. However, their homologues in *N. sylvestris* (the maternal long-day ancestor) regulate flowering under non-inductive short-day conditions by inducing a *FT* homologue.

Overall, the present research studies reinforce the evolutionary differences of *CO* and *COL* genes among plant species, and help to pave the way to precisely incorporate daylength signal into physiological responses that will be of upmost relevance for crop yield.

## Author contributions

FN: Conceptualization, Formal analysis, Writing – original draft, Writing – review & editing. DG: Formal analysis, Supervision, Writing – review & editing. FV: Conceptualization, Formal analysis, Project administration, Supervision, Writing – original draft, Writing – review & editing.
